# 4-Chloro­benzoic acid–quinoline (1/1)

**DOI:** 10.1107/S1600536810046416

**Published:** 2010-11-17

**Authors:** Kazuma Gotoh, Kaori Katagiri, Hiroyuki Ishida

**Affiliations:** a Department of Chemistry, Faculty of Science, Okayama University, Okayama 700-8530, Japan

## Abstract

In the title compound, C_7_H_5_ClO_2_·C_9_H_7_N, the 4-chloro­benzoic acid mol­ecule is almost planar, with a dihedral angle of 2.9 (14)° between the carb­oxy group and the benzene ring. In the crystal, the two components are connected by an O—H⋯N hydrogen bond. In the hydrogen-bonded unit, the dihedral angle between the quinoline ring system and the benzene ring of the benzoic acid is 44.75 (4)°. The two components are further linked by inter­molecular C—H⋯O hydrogen bonds, forming a layer parallel to the *ab* plane.

## Related literature

For related structures, see, for example: Gotoh & Ishida (2007 ▶, 2009 ▶); Ishida & Fukunaga (2004 ▶).
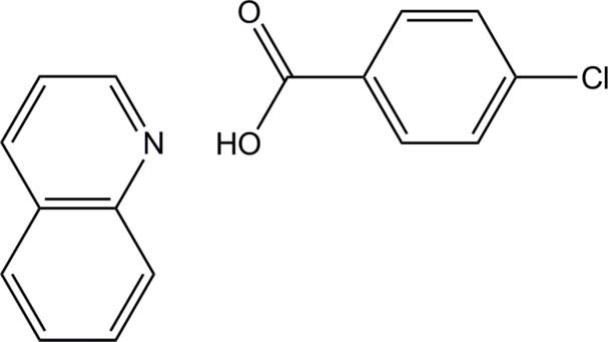


## Experimental

### 

#### Crystal data

C_7_H_5_ClO_2_·C_9_H_7_N
*M* *_r_* = 285.73
Orthorhombic, 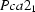

*a* = 13.2385 (5) Å
*b* = 3.8307 (2) Å
*c* = 26.2464 (9) Å
*V* = 1331.03 (10) Å^3^
*Z* = 4
Mo *K*α radiation
μ = 0.29 mm^−1^
*T* = 185 K
0.30 × 0.26 × 0.18 mm

#### Data collection

Rigaku R-AXIS RAPID II diffractometer
Absorption correction: numerical (*NUMABS*; Higashi, 1999 ▶) *T*_min_ = 0.933, *T*_max_ = 0.950
21775 measured reflections
3907 independent reflections
3777 reflections with *I* > 2σ(*I*)
*R*_int_ = 0.017

#### Refinement

*R*[*F* ^2^ > 2σ(*F* ^2^)] = 0.027
*wR*(*F* ^2^) = 0.071
*S* = 1.07
3907 reflections
185 parameters
1 restraint
H atoms treated by a mixture of independent and constrained refinement
Δρ_max_ = 0.29 e Å^−3^
Δρ_min_ = −0.15 e Å^−3^
Absolute structure: Flack (1983 ▶), 1909 Friedel pairs
Flack parameter: 0.03 (4)

### 

Data collection: *PROCESS-AUTO* (Rigaku/MSC, 2004 ▶); cell refinement: *PROCESS-AUTO*; data reduction: *CrystalStructure* (Rigaku/MSC, 2004 ▶); program(s) used to solve structure: *SHELXS97* (Sheldrick, 2008 ▶); program(s) used to refine structure: *SHELXL97* (Sheldrick, 2008 ▶); molecular graphics: *ORTEP-3* (Farrugia, 1997) ▶; software used to prepare material for publication: *CrystalStructure* (Rigaku/MSC, 2004 ▶) and *PLATON* (Spek, 2009 ▶).

## Supplementary Material

Crystal structure: contains datablocks global, I. DOI: 10.1107/S1600536810046416/im2244sup1.cif

Structure factors: contains datablocks I. DOI: 10.1107/S1600536810046416/im2244Isup2.hkl

Additional supplementary materials:  crystallographic information; 3D view; checkCIF report

## Figures and Tables

**Table 1:** Hydrogen-bond geometry (Å, °)

*D*—H⋯*A*	*D*—H	H⋯*A*	*D*⋯*A*	*D*—H⋯*A*
O1—H1⋯N1	0.84 (2)	1.82 (2)	2.659 (1)	176 (2)
C5—H5⋯O2^i^	0.95	2.46	3.159 (1)	130
C8—H8⋯O2^ii^	0.95	2.57	3.252 (2)	129

Symmetry codes: (i) 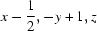; (ii) 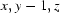.

**Fig. 1.**
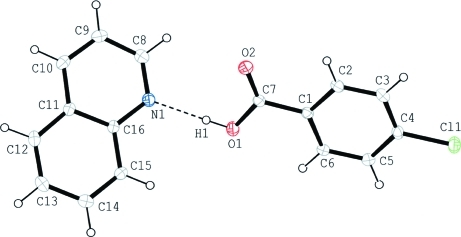
Molecular structure of the title compound, with the atom-labeling. Displacement ellipsoids of non-H atoms are drawn at the 50% probability level. The dashed line indicates the O—H···N hydrogen bond.

**Fig. 2.**
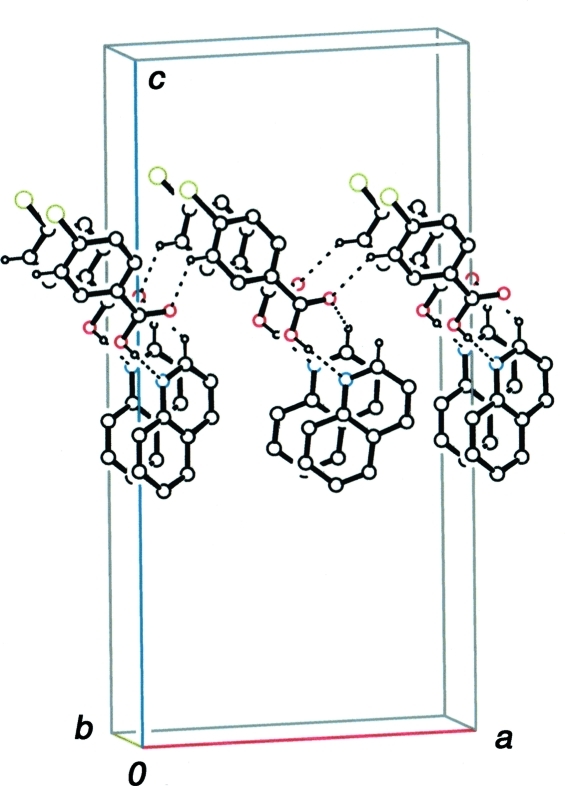
Packing diagram of the title compound, showing the layered structure formed by O—H···N and C—H···O hydrogen bonds (dashed lines). H atoms not involved in the hydrogen bonds have been omitted.

## supplementary crystallographic information

### Comment

The title compound was prepared in order to extend our study on *D*—H···*A* hydrogen bonding (*D* = N, O, or C; *A* = N, O or Cl) in amine–benzoic acid systems (Gotoh & Ishida, 2007, 2009; Ishida & Fukunaga, 2004).

In the crystal structure of the title compound, no acid-base interaction involving proton transfer is observed between the two components, which are are linked by an O—H···N hydrogen bond (Table 1 and Fig. 1). In the hydrogen-bonded unit, the dihedral angle between the quinoline ring system and the benzene ring of the benzoic acid is 44.75 (4)°. The carboxy plane makes dihedral angles of 42.2 (1) and 2.9 (14)°, respectively, with the quinoline ring system and the benzene ring. The two components are further linked by intermolecular C—H···O hydrogen bonds (Table 1), forming a layer parallel to the *ab* plane (Fig. 2). No significant interaction is observed between the layers.

### Experimental

Single crystals were obtained by slow evaporation from an acetonitrile solution (65 ml) of 4-chlorobenzoic acid (156 mg) and quinoline (167 mg) at room temperature.

### Refinement

C-bound H atoms were positioned geometrically (C—H = 0.95 Å) and refined as riding, with *U*_iso_(H) = 1.2*U*_eq_(C). The O-bound H atom was found in a difference Fourier map and refined isotropically. The refined O—H distance is 0.84 (2) Å.

### Crystal data

**Table:**

C_7_H_5_ClO_2_·C_9_H_7_N	*F*(000) = 592.00
*M**_r_* = 285.73	*D*_x_ = 1.426 Mg m^−^^3^
Orthorhombic, *P**c**a*2_1_	Mo *K*α radiation, λ = 0.71075 Å
Hall symbol: P 2c -2ac	Cell parameters from 20625 reflections
*a* = 13.2385 (5) Å	θ = 3.1–30.0°
*b* = 3.8307 (2) Å	µ = 0.29 mm^−^^1^
*c* = 26.2464 (9) Å	*T* = 185 K
*V* = 1331.03 (10) Å^3^	Block, colorless
*Z* = 4	0.30 × 0.26 × 0.18 mm

### Data collection

**Table:**

Rigaku R-AXIS RAPID II diffractometer	3777 reflections with *I* > 2σ(*I*)
Detector resolution: 10.00 pixels mm^-1^	*R*_int_ = 0.017
ω scans	θ_max_ = 30.0°
Absorption correction: numerical (*NUMABS*; Higashi, 1999)	*h* = −18→17
*T*_min_ = 0.933, *T*_max_ = 0.950	*k* = −5→5
21775 measured reflections	*l* = −36→36
3907 independent reflections	

### Refinement

**Table:**

Refinement on *F*^2^	Secondary atom site location: difference Fourier map
Least-squares matrix: full	Hydrogen site location: inferred from neighbouring sites
*R*[*F*^2^ > 2σ(*F*^2^)] = 0.027	H atoms treated by a mixture of independent and constrained refinement
*wR*(*F*^2^) = 0.071	*w* = 1/[σ^2^(*F*_o_^2^) + (0.0482*P*)^2^ + 0.0837*P*] where *P* = (*F*_o_^2^ + 2*F*_c_^2^)/3
*S* = 1.07	(Δ/σ)_max_ = 0.001
3907 reflections	Δρ_max_ = 0.29 e Å^−^^3^
185 parameters	Δρ_min_ = −0.15 e Å^−^^3^
1 restraint	Absolute structure: Flack (1983), 1909 Friedel pairs
Primary atom site location: structure-invariant direct methods	Flack parameter: 0.03 (4)

### Special details

**Table:**

Geometry. All e.s.d.'s (except the e.s.d. in the dihedral angle between two l.s. planes) are estimated using the full covariance matrix. The cell e.s.d.'s are taken into account individually in the estimation of e.s.d.'s in distances, angles and torsion angles; correlations between e.s.d.'s in cell parameters are only used when they are defined by crystal symmetry. An approximate (isotropic) treatment of cell e.s.d.'s is used for estimating e.s.d.'s involving l.s. planes.
Refinement. Refinement of *F*^2^ against ALL reflections. The weighted *R*-factor *wR* and goodness of fit *S* are based on *F*^2^, conventional *R*-factors *R* are based on *F*, with *F* set to zero for negative *F*^2^. The threshold expression of *F*^2^ > σ(*F*^2^) is used only for calculating *R*-factors(gt) *etc*. and is not relevant to the choice of reflections for refinement. *R*-factors based on *F*^2^ are statistically about twice as large as those based on *F*, and *R*- factors based on ALL data will be even larger.

### Fractional atomic coordinates and isotropic or equivalent isotropic displacement parameters (Å^2^)

**Table:**

	*x*	*y*	*z*	*U*_iso_*/*U*_eq_	
Cl1	0.16026 (2)	1.03905 (7)	0.806049 (12)	0.03662 (8)	
O1	0.40632 (7)	0.4457 (3)	0.60147 (3)	0.03433 (19)	
O2	0.53847 (6)	0.6264 (3)	0.64747 (3)	0.03630 (19)	
N1	0.54986 (7)	0.2446 (2)	0.53696 (4)	0.02677 (17)	
C1	0.37402 (7)	0.6999 (3)	0.68200 (4)	0.02308 (18)	
C2	0.41115 (8)	0.8623 (3)	0.72551 (4)	0.02695 (19)	
H2	0.4817	0.9015	0.7289	0.032*	
C3	0.34608 (9)	0.9674 (3)	0.76389 (4)	0.0283 (2)	
H3	0.3713	1.0796	0.7936	0.034*	
C4	0.24300 (9)	0.9056 (3)	0.75818 (4)	0.02659 (19)	
C5	0.20408 (8)	0.7420 (3)	0.71540 (4)	0.0284 (2)	
H5	0.1336	0.7010	0.7123	0.034*	
C6	0.27012 (7)	0.6389 (3)	0.67712 (4)	0.02561 (19)	
H6	0.2447	0.5266	0.6475	0.031*	
C7	0.44796 (8)	0.5884 (3)	0.64212 (4)	0.02529 (19)	
C8	0.63210 (9)	0.1038 (3)	0.55654 (5)	0.0312 (2)	
H8	0.6346	0.0685	0.5923	0.037*	
C9	0.71666 (9)	0.0024 (3)	0.52725 (5)	0.0323 (2)	
H9	0.7742	−0.0989	0.5430	0.039*	
C10	0.71388 (8)	0.0531 (3)	0.47574 (5)	0.0297 (2)	
H10	0.7696	−0.0144	0.4552	0.036*	
C11	0.62751 (7)	0.2070 (3)	0.45316 (4)	0.02400 (18)	
C12	0.61876 (9)	0.2723 (3)	0.40025 (4)	0.0304 (2)	
H12	0.6730	0.2140	0.3781	0.037*	
C13	0.53272 (10)	0.4190 (3)	0.38070 (4)	0.0331 (2)	
H13	0.5275	0.4598	0.3451	0.040*	
C14	0.45165 (10)	0.5100 (3)	0.41306 (5)	0.0314 (2)	
H14	0.3922	0.6106	0.3990	0.038*	
C15	0.45801 (8)	0.4545 (3)	0.46454 (5)	0.0272 (2)	
H15	0.4035	0.5189	0.4861	0.033*	
C16	0.54610 (7)	0.3005 (2)	0.48551 (4)	0.02275 (18)	
H1	0.4522 (17)	0.392 (6)	0.5808 (9)	0.061 (6)*	

### Atomic displacement parameters (Å^2^)

**Table:**

	*U*^11^	*U*^22^	*U*^33^	*U*^12^	*U*^13^	*U*^23^
Cl1	0.04236 (14)	0.03938 (14)	0.02811 (12)	0.00840 (10)	0.00809 (12)	−0.00104 (13)
O1	0.0260 (4)	0.0509 (5)	0.0261 (4)	−0.0014 (3)	0.0017 (3)	−0.0079 (3)
O2	0.0238 (4)	0.0525 (5)	0.0326 (4)	−0.0001 (4)	−0.0006 (3)	−0.0010 (4)
N1	0.0264 (4)	0.0293 (4)	0.0246 (4)	−0.0015 (3)	0.0013 (3)	−0.0015 (3)
C1	0.0232 (4)	0.0249 (4)	0.0211 (4)	0.0006 (3)	−0.0009 (4)	0.0027 (3)
C2	0.0265 (4)	0.0305 (5)	0.0238 (4)	−0.0040 (4)	−0.0027 (4)	0.0023 (4)
C3	0.0356 (5)	0.0272 (5)	0.0221 (5)	−0.0033 (4)	−0.0037 (4)	0.0006 (4)
C4	0.0332 (5)	0.0246 (4)	0.0220 (4)	0.0041 (4)	0.0035 (4)	0.0021 (3)
C5	0.0251 (5)	0.0320 (5)	0.0280 (5)	0.0017 (4)	−0.0015 (4)	0.0004 (4)
C6	0.0238 (4)	0.0303 (5)	0.0228 (4)	0.0014 (4)	−0.0034 (4)	−0.0003 (4)
C7	0.0255 (5)	0.0281 (4)	0.0222 (4)	0.0013 (4)	−0.0006 (4)	0.0038 (3)
C8	0.0330 (5)	0.0319 (5)	0.0286 (5)	−0.0034 (4)	−0.0030 (4)	0.0018 (4)
C9	0.0260 (5)	0.0303 (5)	0.0406 (7)	0.0021 (4)	−0.0066 (5)	−0.0001 (4)
C10	0.0225 (4)	0.0278 (5)	0.0387 (6)	0.0006 (4)	0.0022 (4)	−0.0047 (4)
C11	0.0220 (4)	0.0229 (4)	0.0271 (4)	−0.0034 (3)	0.0022 (4)	−0.0043 (3)
C12	0.0330 (5)	0.0318 (5)	0.0266 (5)	−0.0053 (4)	0.0060 (4)	−0.0050 (4)
C13	0.0427 (6)	0.0319 (5)	0.0248 (5)	−0.0065 (4)	−0.0016 (5)	0.0007 (4)
C14	0.0325 (5)	0.0290 (5)	0.0328 (6)	−0.0008 (4)	−0.0064 (4)	0.0016 (4)
C15	0.0240 (5)	0.0268 (5)	0.0308 (5)	0.0018 (4)	0.0004 (4)	−0.0016 (4)
C16	0.0228 (4)	0.0210 (4)	0.0245 (4)	−0.0028 (3)	0.0019 (4)	−0.0022 (3)

### Geometric parameters (Å, °)

**Table:**

Cl1—C4	1.7434 (11)	C8—C9	1.4123 (18)
O1—C7	1.3194 (14)	C8—H8	0.9500
O1—H1	0.84 (2)	C9—C10	1.3666 (18)
O2—C7	1.2151 (14)	C9—H9	0.9500
N1—C8	1.3194 (15)	C10—C11	1.4162 (15)
N1—C16	1.3681 (13)	C10—H10	0.9500
C1—C2	1.3903 (14)	C11—C12	1.4160 (15)
C1—C6	1.4010 (14)	C11—C16	1.4179 (13)
C1—C7	1.4953 (14)	C12—C13	1.3697 (18)
C2—C3	1.3854 (16)	C12—H12	0.9500
C2—H2	0.9500	C13—C14	1.4125 (18)
C3—C4	1.3931 (16)	C13—H13	0.9500
C3—H3	0.9500	C14—C15	1.3703 (16)
C4—C5	1.3854 (16)	C14—H14	0.9500
C5—C6	1.3891 (15)	C15—C16	1.4180 (14)
C5—H5	0.9500	C15—H15	0.9500
C6—H6	0.9500		
			
C7—O1—H1	108.7 (15)	C9—C8—H8	118.2
C8—N1—C16	118.58 (10)	C10—C9—C8	118.55 (11)
C2—C1—C6	119.79 (9)	C10—C9—H9	120.7
C2—C1—C7	118.11 (9)	C8—C9—H9	120.7
C6—C1—C7	122.08 (9)	C9—C10—C11	119.66 (10)
C3—C2—C1	120.50 (10)	C9—C10—H10	120.2
C3—C2—H2	119.8	C11—C10—H10	120.2
C1—C2—H2	119.8	C12—C11—C10	123.36 (10)
C2—C3—C4	118.78 (10)	C12—C11—C16	118.71 (10)
C2—C3—H3	120.6	C10—C11—C16	117.92 (10)
C4—C3—H3	120.6	C13—C12—C11	120.53 (10)
C5—C4—C3	121.90 (10)	C13—C12—H12	119.7
C5—C4—Cl1	118.89 (9)	C11—C12—H12	119.7
C3—C4—Cl1	119.21 (9)	C12—C13—C14	120.52 (11)
C4—C5—C6	118.74 (10)	C12—C13—H13	119.7
C4—C5—H5	120.6	C14—C13—H13	119.7
C6—C5—H5	120.6	C15—C14—C13	120.52 (11)
C5—C6—C1	120.29 (10)	C15—C14—H14	119.7
C5—C6—H6	119.9	C13—C14—H14	119.7
C1—C6—H6	119.9	C14—C15—C16	119.86 (11)
O2—C7—O1	123.72 (11)	C14—C15—H15	120.1
O2—C7—C1	122.03 (10)	C16—C15—H15	120.1
O1—C7—C1	114.25 (9)	N1—C16—C11	121.59 (9)
N1—C8—C9	123.68 (11)	N1—C16—C15	118.55 (9)
N1—C8—H8	118.2	C11—C16—C15	119.85 (9)
			
C6—C1—C2—C3	−0.61 (16)	C8—C9—C10—C11	0.47 (17)
C7—C1—C2—C3	−179.29 (10)	C9—C10—C11—C12	179.31 (11)
C1—C2—C3—C4	0.35 (16)	C9—C10—C11—C16	−0.50 (15)
C2—C3—C4—C5	0.13 (16)	C10—C11—C12—C13	179.38 (10)
C2—C3—C4—Cl1	−179.61 (8)	C16—C11—C12—C13	−0.81 (15)
C3—C4—C5—C6	−0.34 (16)	C11—C12—C13—C14	0.51 (17)
Cl1—C4—C5—C6	179.40 (8)	C12—C13—C14—C15	0.29 (18)
C4—C5—C6—C1	0.07 (16)	C13—C14—C15—C16	−0.77 (17)
C2—C1—C6—C5	0.40 (16)	C8—N1—C16—C11	0.75 (15)
C7—C1—C6—C5	179.02 (10)	C8—N1—C16—C15	−179.50 (10)
C2—C1—C7—O2	2.21 (16)	C12—C11—C16—N1	−179.93 (9)
C6—C1—C7—O2	−176.43 (11)	C10—C11—C16—N1	−0.11 (14)
C2—C1—C7—O1	−178.14 (10)	C12—C11—C16—C15	0.33 (14)
C6—C1—C7—O1	3.21 (14)	C10—C11—C16—C15	−179.85 (9)
C16—N1—C8—C9	−0.81 (17)	C14—C15—C16—N1	−179.29 (10)
N1—C8—C9—C10	0.20 (18)	C14—C15—C16—C11	0.45 (15)

### Hydrogen-bond geometry (Å, °)

**Table:**

*D*—H···*A*	*D*—H	H···*A*	*D*···*A*	*D*—H···*A*
O1—H1···N1	0.84 (2)	1.82 (2)	2.659 (1)	176 (2)
C5—H5···O2^i^	0.95	2.46	3.159 (1)	130
C8—H8···O2^ii^	0.95	2.57	3.252 (2)	129

Symmetry codes: (i) *x*−1/2, −*y*+1, *z*; (ii) *x*, *y*−1, *z*.
